# Mental Performance and Sport: Caffeine and Co-consumed Bioactive Ingredients

**DOI:** 10.1007/s40279-022-01796-8

**Published:** 2022-11-30

**Authors:** David O. Kennedy, Emma L. Wightman

**Affiliations:** Brain, Performance and Nutrition Research Centre, Northumbria University, Newcastle-upon-Tyne, NE1 8ST UK

## Abstract

The plant defence compound caffeine is widely consumed as a performance enhancer in a sporting context, with potential benefits expected in both physiological and psychological terms. However, although caffeine modestly but consistently improves alertness and fatigue, its effects on mental performance are largely restricted to improved attention or concentration. It has no consistent effect within other cognitive domains that are important to sporting performance, including working memory, executive function and long-term memory. Although caffeine’s central nervous system effects are often attributed to blockade of the receptors for the inhibitory neuromodulator adenosine, it also inhibits a number of enzymes involved both in neurotransmission and in cellular homeostasis and signal propagation. Furthermore, it modulates the pharmacokinetics of other endogenous and exogenous bioactive molecules, in part via interactions with shared cytochrome P450 enzymes. Caffeine therefore enjoys interactive relationships with a wide range of bioactive medicinal and dietary compounds, potentially broadening, increasing, decreasing, or modulating the time course of their functional effects, or vice versa. This narrative review explores the mechanisms of action and efficacy of caffeine and the potential for combinations of caffeine and other dietary compounds to exert psychological effects in excess of those expected following caffeine alone. The review focusses on, and indeed restricted its untargeted search to, the most commonly consumed sources of caffeine: products derived from caffeine-synthesising plants that give us tea (*Camellia sinensis*), coffee (*Coffea* genus), cocoa (*Theabroma cacao*) and guaraná (*Paullinia cupana*), plus multi-component energy drinks and shots. This literature suggests relevant benefits to mental performance that exceed those associated with caffeine for multi-ingredient energy drinks/shots and several low-caffeine extracts, including high-flavanol cocoa and guarana. However, there is a general lack of research conducted in such a way as to disentangle the relative contributions of the component parts of these products.

## Key Points

**Table:** 

Caffeine, when taken alone, is associated with reasonably consistent ergogenic and psychological benefits. However, its effects on mental performance are limited, and do not encompass benefits to several sport-relevant cognitive domains.
Caffeine is typically consumed alongside other bioactive compounds. Evidence suggests that products derived from caffeine-synthesising plants, including high polyphenol cocoa, guaraná and coffee extracts, engender mental performance benefits that exceed those expected from their caffeine content. Similarly, multi-component energy drinks and shots may be more effective than their caffeine content.
Further research disentangling the comparative contributions of caffeine and other co-consumed bioactive ingredients to their combined effects is required in order to inform future recommendations.

## Background

Caffeine is widely consumed in a sporting context. Some three-quarters of athletes consume caffeine before or during competitions, with the highest prevalence amongst endurance sports [1]. Whilst caffeine has well-established ergogenic properties [2, 3], it also exerts a number of purely psychological effects outside of an exercise/sport context. Most prevalent here, caffeine modestly but consistently increases alertness and arousal, alleviating fatigue, and it enhances several aspects of mental performance [4].

Optimal cognitive functioning is essential for peak sporting performance, and it is self-evident that efficient brain function is intrinsic to all forms of sporting performance. For instance, in terms of cognitive domains such as: attention, which incorporates psychomotor function, reaction times and concentration or vigilance; spatial or verbal working memory, which represent the ability to hold small amounts of spatial or verbal information temporarily in mind; and executive function, which incorporates the ability to manipulate information, plan actions and inhibit inappropriate responses. Clearly the comparative contribution of each of these cognitive domains is dependent on the demands of differing sports [5–9]. Indeed, evidence suggests that multiple aspects of an individuals’ mental performance in everyday life, including attention, executive function and working memory, enjoy a positive bi-directional relationship with sporting activities that engage these domains [5–7]. Additionally, modulation of an individual’s subjective psychological state, particularly increased alertness and decreased mental fatigue, will naturally have a knock-on effect on motivation and performance.

In these terms, pure caffeine only offers a very partial option for improving mental performance in a sporting context [10]. Whilst its effects are consistent, they are limited, and caffeine has little to offer in terms of improving spatial or verbal working memory or executive function. It also does not generally have a beneficial impact on long-term memory function. However, caffeine is most often consumed alongside other bioactive compounds, often in products derived from caffeine-synthesising plants. Caffeine enjoys interactive relationships with multifarious other classes of compounds, including many of those that co-exist in the most prevalent caffeinated products. The following therefore describes the mechanisms of action and functional effects of caffeine, and the psychological effects of commonly consumed multi-component caffeinated products. It also assesses, to the extent that the literature allows, whether the non-caffeine components either have relevant independent effects on mental performance beyond those of caffeine, or whether they enjoy an interactive relationship with caffeine that potentiates their own functional effects, or indeed those of caffeine.

## Caffeine Synthesis and Mechanisms of Action

### Ecological Roles and Synthesis

Caffeine, and related methylxanthine alkaloids such as theophylline and theobromine, are ‘secondary metabolites’, phytochemicals that do not play any direct role in the plant’s primary metabolic processes. Instead, they increase the plant’s overall ability to survive by allowing the plant to interact with its environment [11, 12]. In this case, caffeine is synthesised in vulnerable tissue when the plant is under attack by insect or mollusc herbivores or threatened by a range of biotic and abiotic stressors [13, 14]. Once synthesised, caffeine functions as a bitter tasting feeding deterrent [15, 16] and as an insecticide and molluscicide [16, 17]. Additionally, it functions as a neurological behaviour modifier that typically induces a sharp increase in locomotor activity and corresponding reduction in feeding by herbivores [18]. Interestingly, the mechanisms that underlie these interactions with herbivores are largely the same as those that drive caffeine’s effects on human behaviour [12].

The small group of plants that synthesise caffeine include the unrelated, geographically distributed genera of flowering plants that include coffee plants (*Coffea* genus), the kola nut (*Cola acuminata*), tea (*Camelia sinensis*)*,* yerba maté (*Ilex paraguariensis*), guaraná (*Paullinia cupana*) and cocoa (*Theobroma cacao*). The repeated convergent evolution of methylxanthine synthesis in these disparate clades is a reflection of both the utility of caffeine for the plant and the simplicity of methylxanthine synthetic pathways [19, 20].

Caffeine, and the related methylxanthines, are classified as ‘purine’ alkaloids because they incorporate the purine xanthine, which itself is synthesised or recycled in the plant from the ubiquitous purine bases adenine and guanine or their products. One route of synthesis in the plant is directly from the ubiquitous bioactive purine derivative adenosine [13, 21]. Both the ecological roles played by caffeine and its functional effects in humans and other animals are related to caffeine’s structural similarity to adenosine.

### Mechanisms of Action

Adenosine itself is both a ubiquitous bioactive molecule and a building block for a host of other cellular signalling molecules across all forms of life. It therefore plays a wide range of pivotal roles in cellular functioning. For instance it is required for catabolic metabolism in every living cell in the form of the energy carriers adenosine diphosphate/triphosphate (ADP/ATP); it is a component of the ubiquitous second messenger molecule cyclic adenosine monophosphate (cAMP), it is a component of multiple enzymes, including the poly (ADP ribose) polymerase (PARP) family; and, as the adenosine radical adenosyl, it plays pivotal roles in anabolic metabolism in every cell as a component of SAM-e (*S*-adenosyl methionine). Most importantly here, adenosine itself is an inhibitory neuromodulator that serves to decrease overall neuronal activity. In this role it can be released by neurons in a manner similar to that of classical neurotransmitters, but it is most prevalent as a simple breakdown product of cellular adenine nucleotides [22]. Adenosine levels therefore vary with overall brain activity, building up in the cortex and basal forebrain during wakefulness, thus increasing fatigue and decreasing alertness, and then dissipating during sleep [23]. This provides adenosine with a number of properties related to the regulation of tiredness and the sleep–wake cycle, and the homeostatic and neuro-protective down-regulation of brain activity [22, 23].

Orally consumed caffeine is rapidly absorbed and distributed, with peak plasma levels seen at around 30 min post-ingestion, followed by a gradual return to baseline [24]. Although caffeine’s circulating half-life is generally in the region of 3–5 h [25], this can vary depending on a number of factors, including genotype, sex, disease, environmental factors and diet, and the consumption of other bioactive molecules. With respect to genotype, research has been able to identify so-called fast and slow metabolisers of caffeine depending on the cytochrome 450 (CYP450) 1A2 enzyme alleles; with 40% of the population believed to be A/A or ‘fast metabolisers’ and 50 and 10% A/C and C/C ‘slow metabolisers’, respectively [26]. In regard to sex, a recent book [27] summarised the potential for hormonal fluctuations, especially related to oral contraceptive use (and ethinyl estradiol with and without progesterone, in particular) to reduce the plasma clearance and increase the elimination half-life of caffeine. The authors also posit that differences in adiposity could influence the sensitivity to caffeine-induced lipolysis, with females typically having more adipose tissue and less sensitivity, which could underlie metabolic differences between males and females. With respect to disease, slowed metabolism of caffeine is seen in those with liver disease, attributed to reduced N3- and N7-demethylations affecting transformation through the paraxanthine pathway [28], and obesity reportedly increases the volume and distribution of caffeine and prolongs its half-life [29]. The latter review, however, notes the small amount of research here, which was performed over three decades ago in some cases, and the nuance associated with degree of obesity and concomitant pharmacological drug use. With regard to the potential role of other bioactive ingredients, caffeine is metabolised to paraxanthine and to a lesser extent theophylline and theobromine by the action of members of the CYP450 family of enzymes that manage the metabolism and clearance of endogenous and exogenous bioactive compounds [29].

The effects of caffeine within the brain and central nervous system are generally attributed to antagonism of A_1_ and A_2A_ adenosine receptors and the resultant blockade of adenosine’s inhibitory action [30]. This results in decreased fatigue, increased alertness and an increase in the neural activity associated with a variety of neurotransmitters, including dopamine, acetylcholine, noradrenaline, serotonin, glutamate and gamma-aminobutyric acid (GABA) [31]. Adenosine A_1_ and A_2A_ receptors within the vasculature have contradictory effects on vasodilation. Within the brain, the net effect of caffeine’s action at these receptors is the inhibition of adenosine’s vasodilatory effects leading to vasoconstriction and reduced cerebral blood flow [32]. However, in the periphery, the net effect of caffeine is less clear, with contradictory effects on blood pressure and vasodilation, mediated by a complex array of adenosine receptor interactions and other mechanisms (see below) [33]. Subsequent sections below detail how commonly co-consumed compounds, like polyphenols, are also able to influence vascular tone, specifically vasodilation, and these already complex effects from caffeine alone are therefore likely to be even more difficult to interpret in response to co-consumption with other compounds.

Beyond adenosine receptor interactions, caffeine or its metabolites also inhibit the activity of several key enzymes. These include nervous system-specific enzymes that catalyse neurotransmitters, including acetylcholinesterase and monoamine oxidase (leading to increased acetylcholine, dopamine, epinephrine, norepinephrine and serotonin) and amino acid decarboxylase enzymes [34]. Throughout the body, caffeine also inhibits phosphodiesterase, which regulates the cellular secondary messengers cAMP and cyclic guanosine monophosphate (cGMP), and therefore governs cellular responses to other bioactive molecules. It also inhibits several of the poly (ADP-ribose) polymerase (PARP) enzymes that modulate the response to cellular damage, including the modification of inflammatory responses [35–37]. Whilst the physiological effects of some of these individual non-adenosine receptor mechanisms, taken in isolation, might only become relevant at doses of caffeine above those normally found in the diet, their contribution to caffeine’s overall effects on brain function is arguably underestimated [37].

In terms of ergogenic effects, the current consensus is that caffeine’s beneficial effects on physical performance are also largely driven by central and peripheral inhibition of adenosine receptors. The downstream effects of this modulated neurotransmission includes increased motor unit firing, suppression of exercise-related pain, reduced sensation of force, and decreased ratings of perceived physical effort alongside related psychological benefits in terms of reduced fatigue and increased motivation [38, 39]. However, caffeine can also modulate metabolism by interacting with the sympathetic nervous system [40], increasing energy expenditure, thermogenesis and fat oxidation, at least in part via phosphodiesterase inhibition [41]. Caffeine can also modulate the activity of the enzymes responsible for glycogen formation and degradation (i.e., glycogen synthase and glycogen phosphorylase) by interacting with the nucleoside-inhibitor site of glycogen phosphorylase, promoting a reduction of catalytic activity [42]. In skeletal muscles, caffeine also mimics the role of ATP [43], activating ryanodine receptors in the sarcoplasmic reticulum, causing an influx of charged molecules (Ca^2+^) and inhibition of Ca^2+^ reuptake, increasing muscle contraction [37, 44]. One important point to note here is that, while some of the above research included human samples and doses equivalent to those elsewhere in this review (i.e., 3–6 mg/kg), much was predicated on animal models, utilizing what could be regarded as supra-pharmacological doses, and this raises the question of how closely effects can be extrapolated to human samples.

#### Interactive Mechanisms of Action

Given its potential to modulate neurotransmission, enzyme activity and the function of cellular signal pathways, caffeine has potentially wide-ranging modulatory properties with regard to the effects of other bioactive molecules. As an example, co-consumption of caffeine increases both the physiological effects and toxicity of psychostimulant drugs such as amphetamines or cocaine, via both caffeine’s adenosine receptor binding properties and its ability to modulate cell signalling pathways by interacting with the enzyme phosphodiesterase [45].

Caffeine also enjoys multifarious pharmacokinetic interactions that affect the absorption, distribution, metabolism and excretion of many other bioactive molecules. Mechanisms here include the formation of complexes with other acidic compounds; direct gastrointestinal effects including increased diuresis, increased gastrointestinal acid secretion, and accelerated gastric emptying; and modulation of the distribution of molecules, including by increasing the tightness of the blood brain barrier [34]. Caffeine and its metabolite products also compete for access to several ubiquitous CYP450s. Caffeine is primarily metabolised by the CYP1A2 and CYP2E1 enzymes, whilst caffeine’s metabolites (paraxanthine, theophylline and theobromine) are also significant substrates for CYP1A1 and CYP2A6. These enzymes are also involved in the metabolism of a wide range of exogenous nutrients and drugs and many endogenous compounds, including several hormones and neurochemicals that are substrates, inducers or inhibitors of these CYPs. The presence of caffeine and its metabolites can therefore limit the access of these other compounds, or vice versa, to the CYP450 enzymes with which they interact, increasing or decreasing the bioavailability, clearance, effectiveness or toxicity of the compound/drug/nutrient and/or indeed caffeine [34, 46–48]. These same CYP enzymes have also recently been discovered to be active across cognition-relevant brain regions and structures, including neurons [49]. It is therefore likely that caffeine and its metabolites may enjoy a number of un-delineated interactions with a range of neurochemicals [48, 50].

Given its multifarious interactive properties, it is unsurprising that caffeine has significant interactive relationships with a wide range of medicinal drugs, including antiarrhythmics (e.g., mexiletine), bronchodilators (e.g., furafylline and theophylline), oral contraceptives, non-steroidal anti-inflammatory drugs and psychoactive drugs. The latter include antidepressants (e.g., fluvoxamine), antipsychotics (e.g., clozapine), lithium, [34, 46] and natural and synthetic psychostimulants (e.g., ephedrine, amphetamines, cocaine, nicotine) [51, 52]. Clinical trials have also confirmed that caffeine consumption significantly enhances the effectiveness of antidepressant medications [53], increases circulating concentrations of the endogenous neurochemical melatonin [54], increases the bioavailability [34] and effectiveness of aspirin [55], accelerates the absorption of paracetamol [34] and synergistically potentiates the pain-relieving properties of ibuprofen [55, 56]. In combination with ibuprofen, it can also improve the alertness and anxiety of cold sufferers above that seen following either caffeine or ibuprofen alone [57].

Interestingly, instigated by the removal of caffeine from the World Anti-Doping Agency (WADA) list, an analysis of a large sample (> 20,000) of competitive athletes’ urine samples showed that, whilst caffeine levels were significantly lower than the 12 µg/mL threshold set by WADA previously (in all but 0.6% of the sample), nearly 20% of the samples indicated that caffeine had been co-consumed alongside pharmacological drugs that may have interactive properties with caffeine [1]. Examples include some of those examples listed above, such as pseudoephedrine (in 6.4% of samples) and pain killers like paracetamol (1.5%), tramadol (3.5%) and ibuprofen (2.7%), as well as psychoactive compounds like cathine (0.7%). This demonstrates that, whilst the potential for caffeine to incite direct effects on the psychophysiology of athletes alone may arguably have been reduced by this generally low caffeine consumption, the potential for unanticipated interactions with co-consumed pharmacological compounds should not be underestimated.

## Caffeine’s Functional Effects

### Sport and Exercise Performance

Caffeine, when taken by itself, has well-established ergogenic properties. Doses within the optimal range of 3–6 mg/kg body weight have been shown to enhance performance of aerobic endurance exercise, muscular endurance and power, strength, and high-intensity and intermittent exercise [3, 39, 58]. These physical benefits translate into improvements in multifarious sport-specific aspects of physical performance, including those intrinsic to combat, racquet and team sports [39, 59]. However, there is a paucity of studies investigating the ergogenic effects of caffeine at lower doses. A recent review [60] presented evidence that the ergogenic effects of caffeine were largely flat over 3 mg/kg body weight but that they extended down to 2 mg/kg. The authors noted that there was little evidence for effectiveness for doses of caffeine lower than 2 mg/kg, but also scant research assessing the effects of these lower doses.

In general, the caffeine literature here is complicated by unresolved issues such as the role that genetic polymorphisms (e.g., in CYP1A2) [61], habituation [62] and tolerance [63, 64] may play with respect to caffeine’s effects. There is also a pronounced bias against using female participants in ergogenic research [65], although meta-analytic evidence does suggest equivalence between the effects seen in males and females [66], especially in relation to aerobic performance and fatigue [67]. Nevertheless, the latter review did present evidence that aspects of power (e.g., total weight lifted) and speed were more increased in men than women following the same dose of caffeine, meaning potential sex differences in sport-/exercise-specific ergogenic effects of caffeine certainly warrant further attention. The current lack of representation of women in this research area may be due to perceived noise introduced by the hormonal fluctuations of the menstrual cycle. However, recent small-scale trials suggest that this does not have a significant impact. Lara et al. [68], for example, demonstrated that 3 mg/kg caffeine did increase peak aerobic capacity in a cohort of 13 female triathletes (mean age 31 ± 6 years) during the early follicular, preovulation and mid-luteal phase of the menstrual cycle, but the magnitude of this ergogenic effect was similar during all phases. Taken together, ergogenic effects of caffeine appear to be independent of sex and menstrual status but, again, this may not be the case for all types of physical performance/athletes and so should not be wholly disregarded in future trials.

### Psychological Functioning

In terms of purely psychological effects (i.e., outside of a sport/exercise context) it is notable that, in this body of research, caffeine is usually administered at a set dose for all participants, rather than being titrated to an individual’s bodyweight (note: where mg/kg are provided below, an average adult body weight of ~ 70 kg has been assumed). The literature here shows that caffeine, when taken by itself, has consistent effects on cognitive function and arousal/alertness at doses well below those considered to be ergogenic. Here, evidence shows benefits following doses as low as 32 mg (i.e., < 0.5 mg/kg) [25]. Thereafter the cognitive effects of caffeine are readily apparent at 75 mg (~ 1 mg/kg), which is the dose currently required for a European Food Safety Authority (EFSA)-approved claim of caffeine’s psychological effects, but they plateau above this dose. The effects then attenuate as single doses exceed 300 mg [25, 69] or 400 mg [70] (~ 4–5 mg/kg).

Whilst they are consistent, the psychological effects of caffeine alone are restricted to increased subjective alertness/arousal [71–73] and, in terms of mental performance, consistent improvements in the performance of tasks assessing attention or focussed attention/vigilance [72, 74–76]. Caffeine’s effects do not generally extend to other cognitive domains, including several domains potentially relevant to sport. Caffeine in isolation has not been shown to have any demonstrable effect on long-term memory tasks, and has inconsistent effects on working memory and executive function tasks, with evidence suggesting that it can impair the performance of more complex tasks [77–79]. However, a recent meta-analysis [80] does suggest that caffeine’s cognition-enhancing effects may expand into higher-order cognitive functions as a consequence of significant sleep loss or restriction. In this meta-analysis the average period of continuous wakefulness imposed on participants was 31 h, and therefore well beyond the loss of sleep associated with a poor night’s sleep. It is important to note here that, whilst not consistent, a small amount of literature supports anxiety-inducing effects of caffeine at doses consistent with many of the trials described within this narrative review; Loke et al. [81], for example, report increased anxiety with doses of between 3 and 6 mg/kg. However, a review by Smith [82] notes that the data overall are somewhat equivocal here and that the reporting of anxiety by participants is more likely in those who are ‘non-choosers’ of caffeine; i.e., those who do not consume caffeine in their everyday life, thus raising questions about sensitivity, tolerance and habituation, which are now widely believed to be due to genetic polymorphisms of the adenosine A_2_ receptor [83].

### Psychological Functioning During Sport and Exercise

The question of caffeine’s cognitive effects during sport/exercise is somewhat under-researched. A 2021 review and meta-analysis addressing this question [84] only identified 13 studies that fitted the stipulated methodological criteria. However, several of these studies, including one of the five studies subsequently entered into their meta-analysis, involved the administration of multi-component products. Most of the studies also assessed behaviour before and/or after exercise, rather than during exercise. Nevertheless, the findings from this meta-analysis were similar to those from the general literature, in that the cognitive benefits of caffeine were restricted to improved speed and accuracy of attention task performance.

A recent single study also found that attention task performance was improved both during and after exercise following 3 mg/kg caffeine, with greater effects seen for CYP1A2 ‘fast’ metabolisers [85]. However, other studies have generated equivocal evidence as to whether caffeine’s beneficial effects on attention task performance are subsumed within improvement due to simply engaging in exercise alone [9, 86].

Overall, there is a general lack of research assessing the effects of caffeine on cognitive function during active sport or exercise. In part, this is likely due to the complexity of conducting a dual-focus trial where the disruption of the physical activity would be required for the assessment of cognitive function (and vice versa), thus negating their accurate measurement. Additionally, researchers proficient in either physical performance or cognitive assessment are unlikely to be equally proficient in the other domain. This certainly speaks to the need for more interdisciplinary collaboration wherein both areas receive equal focus. One workaround here is to assess cognitive domains required for a particular sport/exercise outside of the physical activity itself (e.g., reaction times in sprint starts), but research is also lacking here. As an exception to this, a recent study reported improved goal-keeper reactive diving times, which were directly related to increased reaction time speed at rest, following 4–6 mg/kg caffeine [87].

It is interesting to note a clear disjunction between the doses of caffeine that are effective in physical, ergogenic terms (optimal dose 3–6 mg/kg), and the doses of caffeine that have an impact on cognitive function and mood (optimal dose 1 to 4 mg/kg). This may be partly explained on the basis that doses of 3 mg/kg or more of caffeine are required to achieve the higher plasma levels required to have the physiological effects in peripheral metabolic tissue that underpin some ergogenic effects, whereas lower plasma concentrations are required to modulate neurotransmission in the brain [69]. Taken together, this might suggest that doses of at least 3–4 mg/kg would be required for both physical and cognitive ergogenic effects to be realised.

## Multi-component Caffeinated Products

Much of the research assessing the effects of caffeine on psychological functioning or sport/exercise performance has involved the administration of pure, anhydrous caffeine [39]. In contrast, caffeine is most often consumed by athletes and the general population in the form of naturally caffeinated drinks, foods and food supplements, or in the form of ‘energy drinks’ and similar products. All of these more ecologically valid sources of caffeine involve the co-consumption of other bioactive compounds, with plant sources of caffeine always containing significant levels of polyphenols. As noted above, at a mechanistic level, caffeine is liable to engender synergistic, additive or modulatory effects when co-consumed with other bioactive compounds. This raises the possibility that the presence of other bioactive compounds may lead to a stronger or broader palette of benefits to mental performance than those that would be expected following caffeine alone. Unfortunately, there is a limited number of studies with the requisite comparator arms to unambiguously disentangle the effects of caffeine from the effects of co-consumed bioactive compounds. However, there is evidence, summarised below, suggesting both that some of the compounds co-consumed with caffeine have independent effects on physiological and brain function, and that they enjoy an additive or interactive relationship with caffeine. Further, this typically results in benefits that are stronger or broader than those following caffeine alone. The evidence summarised below concentrates on energy drinks and the sources of naturally occurring caffeine that represent the most frequently consumed caffeinated products or foods.

### Polyphenols and Caffeine

Caffeine is most often taken in the form of plant-derived caffeinated products, including the most popular sources of caffeine globally: coffee and tea. These plant-based sources of caffeine always naturally contain significant levels of polyphenols. These ubiquitous phytochemicals incorporate within their structure multiple phenyl aromatic hydrocarbon rings with one or more hydroxyl groups attached. The largest group, flavonoids, can be subdivided into chalcones, flavanones and their derivatives the flavones, flavonols, isoflavones, flavanols and anthocyanins [88]. In contrast to the predominant neurotransmission effects of caffeine, polyphenols owe their multifarious health benefits to their global effects on physiological functioning. These are predicated on interactions with, and modulation of, diverse components of a wide range of mammalian cellular signal transduction pathways throughout the body. These pathways, in turn, control gene transcription and a plethora of cellular responses, including cellular metabolism, proliferation, apoptosis, and the synthesis of growth factors, and vasodilatory and inflammatory molecules, which have a direct effect on the metabolic, cardiovascular and inflammatory status of the body [89–91]. In the brain, these cellular signal transduction effects drive modulation of neuro-inflammation, direct and indirect effects on neurotransmission, interactions with neurotrophin receptors and signalling pathways, and increased synthesis of neurotrophins and vasodilatory molecules, leading to increased angiogenesis/neurogenesis and local cerebral blood flow [92–96].

With regard to ergogenic effects, recent meta-analyses suggest that diverse polyphenol rich foods and extracts might accelerate the recovery of muscle function and strength [97, 98], improve post-exercise oxidative and inflammatory status [99], and improve aspects of sporting performance [99, 100]. However, a larger meta-analysis concluded that, when considering polyphenols as a whole, they have a significant but ‘trivial’ effect on endurance exercise performance, with these effects restricted to males in their analysis [101]. However, it is important to note here that effects appear much more nuanced when considering groups of polyphenols separately. While grape, nitrate-depleted beetroot, French maritime pine, Montmorency cherry and pomegranate exhibited ergogenic effects (following both acute and multiple-day supplementation), no significant effects were seen for New Zealand blackcurrant, cocoa, green tea or raisins, and it is likely the relative ineffectiveness of these latter polyphenol groups, on these specific outcomes, that has diluted the overall message of this recent meta-analysis. This speaks to the importance of appreciating the bespoke effects of different polyphenols/polyphenol groups and this is supported by the positive findings reported by other meta-analyses that demonstrate that polyphenols from diverse sources have significant beneficial impacts on cognitive function, including tasks assessing attention, executive function and mental fatigue [102, 103].

Of particular note here, caffeine and polyphenols enjoy a number of potentially additive or interactive relationship effects. These may be due to their common affinity with the same CYP450s, or alternatively caffeine’s effects on the absorption, distribution and clearance of other compounds, or caffeine’s ability to form complexes with other acidic compounds, including phenolics [34]. In line with this, research has demonstrated increased functionality or bioavailability when polyphenols [104–106], or other phenolic compounds [34], are consumed alongside caffeine. As an example, a recent study in humans showed that co-administration of cocoa-flavanols alongside their naturally occurring caffeine/methylxanthines resulted in a synergistic effect on the bioavailability and cardiovascular effects of the cocoa-flavanols [104]. The interactive effects of caffeine can also be seen across the wider literature here. As an example, a meta-analysis of 15 studies showed that, whereas green tea catechins without caffeine had no effect, in combination with caffeine they decreased body weight and/or body mass index (BMI) in comparison to caffeine-matched controls [107]. These interactive effects may underpin the results from a meta-analysis of 120 controlled trials [108] that found that flavanol-rich compound interventions with caffeine (e.g., tea and cocoa extracts) were ranked higher than those without caffeine (apple extracts) in terms of beneficial effects on BMI, waist circumference, total-cholesterol, low density lipoprotein and high density lipoprotein (LDL/HDL)-cholesterol and triglycerides.

#### Cocoa-Flavanols

The rich polyphenol content of cocoa products primarily comprises high levels of the flavanols catechin and epicatechin, and procyanidins, which are oligomers formed from these flavanols. The ultimate level of these phytochemicals is dictated by the fermenting and roasting process [109]. Cocoa also contains low levels of caffeine but higher levels of theobromine. Research generally employs high-flavanol extracts or dark chocolate products with less than 40 mg caffeine. It is unlikely that this amount of caffeine would be exceeded via the consumption of chocolate [110].

Meta-analyses of the substantial body of intervention trial data reveal a consistent beneficial effect of high-flavanol chocolate and cocoa-flavanol extracts on cardiovascular parameters, including inflammatory biomarkers, oxidative stress, gluco-regulation, lipid profiles, blood-pressure and peripheral blood-flow [91, 111–115]. The evidence of ergogenic benefits is less consistent, with some evidence of reduced oxidative stress and modulation of metabolism in a physical performance context, but no consistent evidence of improved exercise performance [116–118].

In terms of psychological function in an exercise context, two small studies have assessed the brain function effects of cocoa-flavanols at rest and after exercise. In the first, cerebral blood flow was assessed in the prefrontal cortex via near-infrared spectroscopy (NIRS). Here, cocoa-flavanols increased cerebral blood-flow (specifically, oxygenated haemoglobin) during the single cognitive task only prior to exercise, with exercise itself also increasing cerebral blood-flow (oxygenated, deoxygenated and total haemoglobin), improving reaction times and engendering an increase in neurotrophic factors post-exercise [119]. It is important to note, however, that whilst NIRS provides a measure of blood flow in the top layers of cortical tissue, it is unable to measure more deeply than this. Additionally, NIRS is not considered a traditional tool in the assessment of brain perfusion (i.e., the measurement of blood taken up by specific brain regions; e.g., positron emission tomography (PET) or magnetic resonance imaging (MRI)), although many regard the modulation of deoxygenated haemoglobin as a reliable indicator of this in the same way that it denotes the blood oxygen level dependent (BOLD) signal in functional MRI (fMRI). However, utilization of imaging techniques such as fMRI/PET is not possible during the assessment of physical performance, so NIRS presents a practicable alternative here. Finally, a subsequent study reported improved executive function task performance before and after exercise as a consequence of consuming a high versus low cocoa-flavanol drink, with no interaction with exercise [120].

In terms of brain function in a wider, non-sport/exercise context, cocoa-flavanols consistently increase cerebral blood-flow and engender the synthesis of neurotrophic factors such as brain-derived neurotrophic factor (BDNF) [121]. Single-dose, crossover trials comparing high versus low cocoa-flavanol extracts have demonstrated reduced mental fatigue and improved cognitive function during cognitively demanding tasks [122], and tasks that assess attention [123, 124] and spatial memory [123]. Longer-term supplementation with cocoa-flavanol extracts for 4 weeks also increased attention and executive function task performance, alongside beneficial effects on multiple biomarkers of health, in 90 heathy elderly [125] and 90 sufferers from age-related cognitive impairment [126].

The benefits of chocolate are less clear and suffer from a smaller literature. A recent study demonstrated long-term memory benefits 2 h after high-flavanol dark chocolate when compared to white chocolate, although comparative caffeine levels in the interventions were not reported [127]. Conversely, in older adults, 8 weeks’ administration of high-flavanol chocolate failed to improve executive function task performance in the healthy elderly [128]. However, it should be noted that the low flavanol control intervention still contained a reasonably high level of the putative active ingredient (86 vs. 410 mg flavanols). This raises the issue of not having a true control in such realistic intervention trials, a trade-off that is practically impossible to resolve, and the additional concession to trial design in having either a crossover trial (which would be the gold standard) where double-blinding (again, the gold standard) is not achievable, or a between-subjects design, to accommodate this inability to mask treatment, which then injects the potential for individual differences to buoy one treatment condition above the other/s.

Taken as a whole, a recent systematic review of this literature concluded that acute or chronic administration of cocoa-flavanols most reliably enhanced executive function and memory and decreased task-related mental fatigue [129]. This was supported by a meta-analysis of only chronic supplementation studies (2–3 months), which also reported improvements in executive function task performance [130]. A complementary meta-analysis of the mood effects of acute and short-term high flavanol cocoa studies reported improved depression, anxiety and positive affect following high cocoa-flavanol interventions [131]. This summary comes with a caveat, however. Much of the cardiovascular and psychological research here has used high cocoa-flavanol extracts with low levels of caffeine and compared them to caffeine-matched low-flavanol controls. This research therefore delineates the effects of the higher doses of flavanols over and above any effects of their caffeine content and, therefore, runs the risk of underestimating the effects of the cocoa-flavanol caffeine combinations.

In conclusion, and bearing the last point in mind, the evidence to date does suggest that cocoa-flavanols exert beneficial effects on mental performance that are much broader than those expected from their caffeine content alone. The extent to which this represents an interactive effect between caffeine and the polyphenol content, rather than being solely predicated on the latter, remains to be explored.

#### Guaraná (*Paullinia cupana*)

The phytochemistry of guaraná seed extracts has some similarities to cocoa, with significant levels of the flavanols epicatechin and catechin, and procyanidin and proanthocyanidin flavanol oligomers [132]. Extracts also contain several triterpene compounds. However, guaraná’s purported ergogenic and stimulant properties are often attributed to the presence of naturally occurring caffeine, which typically comprises 2.5–5% of a crude extract’s dry-weight, depending on extraction and manufacturing processes [133, 134].

Whilst there is little methodologically adequate research investigating the effects of guaraná on physical performance, two single-dose, crossover studies have assessed its cognition and mood-enhancing properties in an exercise/sport context. In the first of these, single doses of a product combining guaraná extract (40 mg caffeine or ~ 0.6 mg/kg) and multivitamins improved working memory and episodic memory task performance both before and after 30 min of treadmill running in 40 young males. The guaraná intervention also decreased ratings of perceived effort during the exercise [135]. Subsequently, in a comparatively small study (*n* = 10), consumption of a product containing guaraná extract with 60 mg caffeine (< 1 mg/kg) was equally as effective as 200 mg of caffeine (~ 3 mg/kg) in decreasing perceived exertion during treadmill running and in enhancing the speed of performing a single attention/executive function task after the exercise [136]. The guaraná condition also exceeded the effects of the much higher dose of caffeine in terms of improved cognitive task accuracy.

In a non-sport/exercise study, the guaraná/vitamin product utilised above [135] engendered benefits to cognitive function that were greater than would be expected from the low dose of caffeine in the product. The product improved both the speed and accuracy of a demanding focussed attention task and reduced mental fatigue during extended performance of cognitively demanding tasks [137]. Subsequently, a brain-imaging study [138] confirmed the mental performance-enhancing properties of the same product and demonstrated physiological modulation of brain function using fMRI.

Several, acute, placebo-controlled, crossover studies have also confirmed that caffeine is not the principal psychoactive component of guaraná extracts. In the first of these, 75 mg of guaraná extract containing very low levels of caffeine (9 mg or ~ 0.12 mg/kg) resulted in improved performance of attention, episodic memory and working memory/executive function tasks across the 6-h post-dose testing period [133]. A subsequent dose-ranging study found that doses of the same extract containing negligible caffeine (4.5 mg or < 0.1 mg/kg) could improve episodic memory task performance and ratings of contentedness for 6 h post-dose. In this study only, the highest dose of guaraná extract, containing 35 mg caffeine, increased ratings of alertness [134]. Finally, in perhaps the clearest study, a study compared a product combining guaraná extract and multivitamins directly to its caffeine content (100 mg). This study demonstrated improvements in attention task performance following the guaraná/multivitamin condition that were significantly greater than following both placebo and the equivalent dose of caffeine alone [139].

In conclusion, these studies demonstrate that guaraná extracts are associated with broader and stronger improvements in mental functioning than their caffeine content alone would warrant. Further, these psychological benefits are seen at doses of guaraná that include quantities of caffeine well below psychoactive levels. Whilst this confirms the independent effects of guaraná’s non-caffeine components, the interactive contribution of low-doses of caffeine to these effects has received little attention and so it is not possible to summarise this here.

#### Coffee (*Coffea* Genus) Polyphenols

*Roasted coffee* The process of roasting coffee leads both to the creation of novel compounds and the conversion of existing compounds. This gives roasted coffee a particularly complex phytochemistry. Despite the deleterious effects of roasting, the predominant non-methylxanthine phytochemicals in coffee are polyphenolic chlorogenic acids (CGA), alongside several simple phenolic acids and their derivatives [140]. The process of decaffeinating coffee also affects these polyphenol levels; however. Olechno et al. [141], for example, report the total phenolic content (as gallic acid equivalent) as reaching ~ 650 mg in ground Arabica coffee but only ~ 400 mg in the decaffeinated alternative. This should be taken into consideration when interpreting the effects of decaffeinated coffee discussed below.

A recent umbrella review of caffeine meta-analyses [142] noted that, whilst roast coffee is often used as a source of caffeine by athletes and non-athletes, it is rarely used in research assessing the ergogenic effects of caffeine. As such, the results of the small body of research that has directly compared coffee and caffeine are somewhat equivocal [143]. The situation is similar with regard to psychological functioning, where there are no relevant studies in a sport/exercise context. More generally, whilst coffee has often been compared to a decaffeinated coffee control, purportedly to assess the effects of caffeine, there is a lack of research employing the requisite comparator arms to disentangle the effects of caffeine from those of the other bioactive components; an important consideration bearing in mind the potential reduction in polyphenol levels noted above. One recent study though did compare the cognitive and mood effects of caffeinated and decaffeinated coffee to an inert coffee-flavoured placebo [144]. The results showed that both the caffeinated and the decaffeinated coffee drinks led to increased alertness, but that the caffeine-containing drink alone evinced significant cognitive effects. However, the overall pattern of results showed that the decaffeinated drink fell between the placebo and caffeinated drink on most measures, leading the authors to surmise a modulatory effect of the non-caffeine components of coffee. However, one must also consider the anticipatory effects of consuming a coffee drink, with most people aware of the psychophysiological effects of the caffeine component and perhaps even subjectively detecting (or, alternatively, expecting) its presence or absence in the investigational drink.

*Green coffee* Unroasted ‘green’ coffee benefits from retaining much higher levels of CGA. The consumption of high CGA coffee, made by combining roasted and green beans, for 8 weeks has previously been shown to have beneficial effects on multifarious cardiovascular parameters [145, 146]. For example, two acute dosing studies have demonstrated improved postprandial hyperglycaemia and vascular endothelial function following high CGA coffee in comparison to a caffeine-matched placebo [147, 148]. However, a single physical exercise study found that a light roast high-CGA/caffeine coffee (1066 mg/474 mg, respectively) improved overall mood, but was no more effective than a dark roast low CGA/caffeine (187 mg/33 mg, respectively) coffee in terms of cycling performance or post-exercise oxidative stress and inflammatory status [149]. With such a small literature, however, it is not possible to draw very firm conclusions here and, as will be seen in subsequent sections, the effects of caffeine and/or its co-consumed compounds can often be very activity/sport specific. As such, cycling may not be benefited here but, again, with just one trial, this conclusion is probably premature.

With regard to brain function, one crossover study [150] showed that a decaffeinated high-CGA green coffee (521 mg CGA, 11 mg caffeine) improved the performance of attention tasks, subjective alertness and other aspects of psychological state in 39 healthy older adults, whereas a standard decaffeinated instant coffee (224 mg CGA) had no effect. A subsequent study [151] replicated the beneficial psychological effects of the decaffeinated high-CGA coffee, but found that neither a placebo drink nor a control drink, containing the chlorogenic acid and caffeine components, had any effects. Taken together, these studies show that a low caffeine, high CGA green coffee has beneficial psychoactive effects, but that these effects may depend in part on interactions with components other than just caffeine and CGA.

Two studies have also assessed the effects of caffeine-free green coffee extracts. The first, a parallel-groups study, measured cognitive function in 38 middle-aged/older adults who reported subjective memory decline. The results showed that, after 16 weeks’ supplementation, a drink containing 300 mg CGA had cognitive effects limited to a psychomotor task and an executive function task, in comparison to placebo. Effects were not seen earlier than this (i.e., at the 8-week assessment point) [152]. In a follow-up, crossover study in 34 individuals with mild cognitive impairment, 12 weeks’ administration of a similar high CGA (550 mg) beverage resulted in improved performance on a single executive function/attention task [153]. Together, these findings might suggest that a minimum of 12 weeks is required to exert effects on these cognitive performance outcomes.

*Coffee berry* A small but recently growing body of research has also investigated the behavioural effects of coffee berry extracts made from the fruit pulp surrounding the coffee bean. These extracts are particularly high in chlorogenic acids and a typical dose delivers levels of caffeine similar to decaffeinated coffee (~ 1 to 2% caffeine). To date, there has been little research investigating the ergogenic effects of coffee berry, although one small study found that this intervention caused an improvement in antioxidant status but had no effect on exercise parameters [154].

In a non-exercise context, single doses of coffee berry extracts have been shown to increase the synthesis of neurotrophic factors such as BDNF [155–157], and a range of coffee berry extract doses (100, 300, 1100 mg) reduced the mental fatigue, and attenuated the decreased alertness, associated with extended performance of demanding cognitive tasks [158, 159]. A follow-up study contrasting the cognitive and psychological state effects of 1100 mg coffee berry extract alone, and combined with apple polyphenol extract, found that coffee berry alone increased alertness and vigour and decreased fatigue across the 6 h of post-dose assessments. However, this effect was blunted by the addition of apple polyphenols, although this extract did improve the performance of an executive function task [160], demonstrating the importance of, where possible, considering the effects of treatment arms in isolation. Brain imaging studies have also demonstrated that drinks containing coffee berry extract (1100 mg) can increase cerebral blood flow in the frontal cortex during cognitive tasks [159] and increase functional connectivity between brain regions implicated in task performance [157].

One study has also investigated chronic effects. In this case, when coffee berry extract was taken in the morning or twice per day for 7 and 28 days by sufferers from mild age-related cognitive impairment, there was a significant improvement in speed and a trend towards improved accuracy on a demanding working memory/executive function task [161]. This effect was not seen when the extract was only taken in the evening and may speak to the benefits of the alerting effects of coffee berry in the morning when the psychophysiological effects of this are more impactful (i.e., the daytime period comprises tasks that require increased arousal, as opposed to the evening and overnight) and welcomed (i.e., participants actively desire the subjective and objective increase in alertness in the morning when rousing from sleep). This also fits with the peak plasma levels of caffeine, which would be anticipated between 15 and 30 min in most consumers and may adversely incite wakefulness when coffee berry is consumed in the evening.

In conclusion, whilst little research has been conducted in such a way as to disentangle caffeine’s effects, evidence does suggest that coffee’s non-caffeine phytochemical components exert some independent effects on psychological functioning and may modulate caffeine’s effects, or vice versa. Although neither green coffee nor coffee berry products have benefitted from substantial research efforts as yet, both contain higher levels of potentially bioactive CGAs, and might be expected to exert greater independent effects on function. It is also possible that the functionality of these low caffeine extracts might be increased by higher levels of caffeine.

#### Green Tea (*Camellia sinensis*)

Green tea contains significant levels of flavanols, including catechin, epicatechin and the tea-specific polyphenol epigallocatechin gallate (EGCG). It also contains the tea-specific amino-acid ʟ-theanine and caffeine. Meta-analyses of controlled trial data show that the consumption of green tea extracts is associated with a number of cardiovascular and anthropomorphic benefits, including enhanced total antioxidant status [162], improved glucoregulation [163] and significant benefits to weight, BMI and waist circumference, irrespective of caffeine content [164]. Whilst the exercise performance effects of green tea extracts remain unclear, consumption of caffeinated green tea extracts for more than 1 week has been shown to reduce exercise-induced oxidative stress [165].

To date there is little research assessing the brain function effects of green tea extracts or tea catechins, and no studies directly assessing brain function in a sport/exercise context. Two fMRI studies demonstrated modulation of brain function following single doses of a whey milk drink supplemented with green tea extract [166, 167], but failed to match their whey control drink for caffeine. Two studies also demonstrated cerebral blood flow and electroencephalogram (EEG) effects of single doses of the tea polyphenol EGCG [122, 168], but in the absence of any cognitive performance effects.

There are rather more data with regard to the green tea components caffeine and ʟ-theanine and their potential interactions. Whilst ʟ-theanine by itself is not associated with any significant benefits to mood or cognitive function, a meta-analysis of the data from seven acute dose studies found that caffeine and ʟ-theanine combinations increased alertness and attention task performance for the first 2 h after consumption. The disparate doses employed in these studies ranged from 30 to 250 mg caffeine and from 12 to 200 mg theanine, with a stronger relationship between caffeine dose and performance of the two [169].

Several studies have also directly investigated the potential interactive effects of caffeine and l-theanine. One study [170] found that a single dose of 50 mg caffeine had its expected effects in terms of improved alertness and increased accuracy on an attention task, but that the combination of caffeine with 100 mg ʟ-theanine resulted in additional benefits in terms of improved attention task performance and improved long-term memory, an outcome not typically associated with caffeine alone. A subsequent study [171] found that whereas caffeine (150 mg) and caffeine combined with ʟ-theanine (250 mg) elicited common improvements in the performance of a rapid visual information processing (RVIP) task and decreased subjective ‘mental fatigue’, the caffeine/ʟ-theanine combination also led to a number of significant benefits over those seen following caffeine alone, including improved alertness and tiredness and enhanced working memory performance; again, this was an outcome not typically associated with caffeine alone. Similarly, whilst single doses of 200 mg of ʟ-theanine and 160 mg of caffeine improved the performance of one of two attention tasks, their combination resulted in a numerically more significant effect than either treatment alone [172]. In contrast to these previous studies, a further study [173] found that whereas both 200 mg caffeine and 200 mg ʟ-theanine had significant but markedly different effects on attention task performance, their combination had no cognitive effects. In this instance, caffeine both alone and in combination with theanine modulated mood, but theanine alone had no effect. Similarly, a further study showed that a low dose of ʟ-theanine (75 mg) attenuated some of caffeine’s (50 mg) cognitive and mood effects [174]. This study also found that the reduction in cerebral blood-flow in the frontal cortex during task performance caused by caffeine was abolished by the addition of ʟ-theanine. Finally, a recent brain imaging (fMRI) study showed that whilst both ʟ-theanine (200 mg) and caffeine (160 mg) exerted different, independent effects on brain activation, the two compounds taken together elicited a synergistic, interactive effect on activation in brain regions associated with task performance [175].

In conclusion, given the global popularity of teas, the effects of green tea extracts/infusions are comparatively under-researched. There is evidence that caffeine increases the bioavailability of tea flavanols [105, 106] and evidence of synergistic relationships between caffeine and the tea amino-acid ʟ-theanine with respect to brain function. The mental performance effects of tea extracts or infusions and the interactive contributions of their caffeine, ʟ-theanine and flavanol components deserve greater attention. Additionally, the delivery of caffeine in its naturally consumed state within tea and coffee drinks arguably offers a much more realistic insight into its effects than an isolated, encapsulated dose of caffeine. However, this raises the question of whether additions of milk and sugar, in particular, are permitted. These may make the drink more palatable for many consumers, but may also alter the plasma kinetic profile of phenolics. Zhang et al. [176], for example, reported that carbohydrate and fat can increase the absorption and time to reach maximum concentration (*t*_max_) of co-consumed polyphenols, and so it is unsurprising that Renouf et al. [177] observed that the addition of creamer and sugar to black coffee significantly reduced the concentration maximum (*C*_max_) and *t*_max_ of chlorogenic acids. Further, a small amount of research suggests that this can negatively impact some of the mechanisms relevant to this review; Lorenz et al. [178], for example, report a blunting of positive vascular effects, induced by tea, with the addition of milk. As a result, the findings of studies that permitted the use of milk and sugar should likely be considered differently to those trials that administered black coffee alone. Moving forwards, it is important for future trials to decide whether the trade-off between having a more palatable investigational product, especially with older participants, outweighs the benefits of having a macronutrient-free caffeine drink.

### Energy Drinks and Shots

Caffeinated energy products include a wide range of gels, bars and drink powders. However, ready-made energy drinks and shots have lately attracted the majority of relevant, product-specific research and it could be argued that this is due to the fact that these products still dominate the market. More recently, functional chewing gums containing caffeine have entered the market, and a meta-analysis published this year [179], comprising 14 studies and over 200 participants, reported a significant ergogenic effect of caffeine gum, relative to placebo, especially for trained individuals, in both endurance and strength/power activities. This was evinced when consumed within 15 min of commencing the activity and at doses of 3 mg/kg and above only. However, to the best of our knowledge, no functional caffeinated gums containing additional compounds (even glucose) have yet been investigated with randomised controlled trials, and so the effects here are exclusively attributed to caffeine. As such, caffeine-only gums do not fall within the purview of this review.

Energy drinks and shots typically contain caffeine and taurine, often in combination with glucose, amino acids, vitamins or herbal extracts. However, it is a common practice to simply attribute all of the effects of energy drinks/shots to their caffeine content alone, even if this attribution is not based on any evidence (i.e., the trial does not include a caffeine-only arm). Conversely, meta-analyses purportedly investigating the ergogenic effects of caffeine have often conflated pure caffeine and energy drink studies (e.g., [59, 66]), ignoring the likely key role that caffeine plays within the drink matrices. In reality, there is increasing evidence of interactive effects between caffeine and the other bioactive components of these products.

In terms of ergogenic effects, a recent meta-analysis of the data from 34 studies [180] found that energy drinks containing caffeine and taurine resulted in significantly improved endurance exercise test performance, jumping, muscle strength and endurance, and cycling and running performance. Importantly, these effects were evident from doses of caffeine (~ 1 mg/kg) that were lower than those that would typically be considered ergogenic [39]. The benefits following the energy drinks were also significantly related to the amount of taurine in the drinks rather than caffeine. These findings suggest that taurine plays a pivotal role in the effects of products combining caffeine and taurine, and finds support in a subsequent meta-analysis confirming the ergogenic effects of taurine mono-treatments [181]. However, a more recent review suggests that the effects of taurine alone, in the absence of caffeine, are equivocal [182]. Whilst this review of 19 trials did observe positive effects of taurine supplementation across a range of activities (*V*O_2max_, time to exhaustion, 3- and 4-km time-trial, anaerobic performance, muscle damage, peak power and recovery), this appeared hugely buoyed by timing of ingestion and the type of exercise protocol. The ergogenic effects of taurine were most effective at 1–3 g/day, when consumed over sub-chronic dosing regimens of 6–15 days, and where doses were consumed 1–3 h prior to activity. Given that plasma taurine concentrations peak at approximately 1-h post oral consumption, it is likely that the above acute ergogenic effects are due to mechanisms unrelated to muscular changes but rather directly related to effects within the central nervous system.

It is also likely that glucose plays a pivotal role in caffeinated energy drinks above and beyond the effects of caffeine, or indeed glucose, in isolation. Carbohydrate ingestion has a well-established ergogenic effect on endurance exercise [183] and, more recently, resistance exercise performance [184], and so it is unsurprising that a recent meta-analysis of energy drinks, containing both caffeine and glucose, observed similar ergogenic benefits across a range of exercise types [185]. These included cycling, power-based activities (including within team sports) and more fine-motor abilities like serving and strokes in racket sports and performance in golf and fencing. The effects appeared fairly nuanced, depending on the sport, but 3 mg/kg was the minimum dose required and consuming this prior to long-duration exercise produced the most positive results. The authors raise the interesting point here that consumption under these conditions serves to both supplement ergogenic compounds like caffeine and glucose, as well as to rehydrate. As such, any psychophysiological effects of these compounds could not be disentangled from the effects of hydration alone, a function which, in itself, has a huge impact on endurance exercise in particular [186].

There are very few studies specifically investigating the effects of energy drinks on psychological functioning in a sport/exercise context. However, an energy drink (comprising 200 mg caffeine, taurine, carbohydrate, amino acids) improved performance on a 35-km cycling time-trial, and enhanced attention/psychomotor function tasks measured before and after the exercise, in non-caffeine-withdrawn athletes [187]. A recent study comparing an energy drink to an isocaloric control drink also demonstrated ergogenic benefits plus improved performance on a simple reaction task that was interposed between warm-up and a bout of maximal exertion. These effects were seen alongside improved mood, vigour and ratings of perceived effort measured post-exercise [188]. In contrast, two studies failed to establish any energy drink-related benefits to cognitive task performance following physical exercise [189] or a session of eSports [190], although this is most likely to be due to the very small samples employed.

It should also be noted that the sport/exercise studies noted above only employed cognitive tasks sensitive to caffeine. This therefore leaves open the question of whether the combination with other bioactive ingredients resulted in broader effects than those expected following caffeine alone. In this respect the non-sport/exercise literature is more helpful. Taken as a whole, caffeine-containing energy drinks have consistent beneficial effects on attention task performance [191]. Studies comparing energy drinks to an isocaloric glucose containing placebo have also demonstrated improved simulated driving performance [192] and benefits that would not be expected from caffeine alone, including improved memory performance [193] and enhanced working memory in the absence of improved attention [194]. A particularly thorough crossover study, involving a large sample (*n* = 94) of healthy adults, compared a carbohydrate-free energy shot to placebo over 6 h post-dose. The results demonstrated broad cognitive benefits that included improved accuracy and speed of attention task performance and improved alertness. More importantly, improvements were also seen on measures that would not be sensitive to caffeine, including across working memory and episodic memory tasks and in ratings of depression and anxiety. All of these improvements were also seen during the later assessments, when the effects of caffeine might be expected to be waning. A subsequent, smaller and less methodologically stringent study broadly confirmed the findings of this trial and demonstrated that the effects of the same energy shot were broader and more pronounced than either caffeine alone or coffee with a similar level of caffeine [195].

With regard to specific ingredients, two studies have compared caffeine, taurine and their combination. In one of these studies taurine attenuated the increased alertness associated with caffeine alone [196], and in the other taurine blunted the increased speed of attention task performance associated with caffeine [197]. However, it should be noted that these two studies used a very limited selection of caffeine-sensitive attention tasks, and do not preclude the possibility that caffeine/taurine might have had unmeasured interaction effects in terms of alternative cognitive domains that are not sensitive to caffeine. Irrespective of the direction of the functional relationship seen here, these results also confirm that both taurine and caffeine contribute to the effects of products that combine them. From a mechanistic perspective, the competition between caffeine’s metabolites and taurine for access to the common CYP450 enzyme that metabolises them [198] could theoretically contribute to any modulation of caffeine’s or taurine’s effects in either direction. Interestingly, a cocktail of herbal extracts, several phytochemical components of which are common substrates for caffeine’s CYP450s, also attenuated the beneficial cognitive and mood effects of caffeine [199].

Overall, there is no evidence to support the contention that the psychological effects of energy drinks are solely attributable to their caffeine content. In contrast, the small amount of available evidence suggests that multi-component energy drinks and shots will have beneficial physical and psychological effects that are either stronger, or in the case of cognition, broader, than would be expected from their caffeine content alone.

## Other Phytochemicals (Non-caffeinated)

In terms of potential benefits to mental performance, a number of phytochemicals and herbal extracts derived from non-caffeinated plants engender mental performance benefits that are broader than those seen following caffeine. Whilst these extracts are most often consumed by themselves, several are commonly found in functional drink products. However, the levels of bioactive components in the extracts used in energy drink products are unclear, and no research has attempted to disentangle any interactions with caffeine. The following is a brief summary of evidence regarding some potential candidates for enhancing mental performance.

### Polyphenols

Two ‘atypical’ polyphenols have garnered potentially relevant evidence suggesting mental performance benefits: curcumin and mangiferin. Meta-analyses of early data suggested that curcumin, the principal polyphenol in turmeric, may be effective in treating mood disorders [200]. Of relevance here, more recent evidence showed that both a single dose [201] as well as 4 weeks’ [201, 202] and 12 weeks’ [202] administration of an enhanced bioavailability curcumin (400 mg) engendered improvements in attention and working memory, with additional improvements in mental fatigue or alertness seen after 4 weeks. After 12 weeks’ administration these improvements also extended to the performance of cognitively demanding tasks.

Another potential candidate is mangiferin, the principal polyphenol in mango leaf extracts. A number of studies have demonstrated acute and chronic ergogenic benefits of a mango leaf extract comprising 60% mangiferin administered in combination with luteolin or quercetin [203–206]. A recent single-dose study extended these findings to brain function [207] and demonstrated wide-ranging improvements to overall accuracy of cognitive task performance, including specific benefits to attention, memory and executive function tasks, across 6 h post-dose, following the consumption of 300 mg of the same mango leaf extract. The performance of cognitively demanding tasks was also improved.

### Monoterpenes

Volatile terpenes, which comprise the principal component of essential oils, have a number of significant direct (e.g., receptor binding) and indirect (e.g., enzyme inhibition) effects on neurotransmission. The beneficial psychological effects of single oral doses of encapsulated monoterpene rich sage (*Salvia officinalis/lavandulaefolia*) essential oils, in comparison to placebo, include demonstrations of improved long-term memory task performance [208, 209], working memory/executive function [210], and improved attention task performance and alertness, alongside reduced mental fatigue during the performance of mentally demanding tasks [209]. Similarly, an oral single dose of encapsulated peppermint (*Mentha piperita*) essential oil resulted in improved performance of cognitively demanding attention/executive function tasks, alongside reduced mental fatigue [211]. Volatile terpenes are readily absorbed by mucosal membranes, and in a later study, the wearing of a peppermint infused non-transdermal skin patch for 6 h resulted in improvements in memory, attention and alertness in comparison to a non-aroma skin-patch in young adults [212].

### Diterpenes

Extracts of *Ginkgo biloba* leaves contain high levels of both diterpenes (ginkgolides/bilobalide) and polyphenols. Research demonstrates cognitive enhancement, including in terms of memory and attention task performance, following single doses of ginkgo extract [213–218] in young adults, and following supplementation for 7 days or longer in both younger [219] and older [220–222] participants. Across these trials, doses ranged between 30, 120, 150, 180, 240, 300 and 600 mg/day. The latter includes two sizeable studies in middle-aged participants that demonstrated improved memory and attention performance after 6 weeks’ supplementation [223, 224]. These latter trials were performed by the same research team (as were [216, 217] and [221, 222]), and this may contribute to the relatively clear pattern of effects attributed to *Ginkgo biloba*, relative to many of the other compounds covered within this review. In all three cases, key methodological principles, such as dose and the source of the investigational product, were maintained between trials and this allows for a more robust comparison across the field. It is rarely possible for one research team to develop a consistent research profile with just one compound like this, but it would be prudent for disparate teams to try, where possible, to align methodological practices and make it easier to compare effects across the literature.

### Triterpenes

The primary bioactive component of Asian (*Panax ginseng*) and American ginseng (*Panax quinquefolius*) extracts are triterpene ginsenosides. Compounds from this class owe their bioactivity to a structural similarity to many animal hormones. Single doses, ranging between 100 and 960 mg of Asian ginseng are associated with improved accuracy of memory tasks [133, 225–228], increased speed of attention task performance [133, 229], and concomitantly improved performance and mental fatigue during performance of cognitively demanding executive function/attention tasks [230, 231]. Single doses of a standardized American ginseng extract also resulted in improved working memory and dose-dependent increases in speed of task performance [227] and improved working memory performance [228]. Again, the consistency of these effects can partly be attributed to methodological consistency across many of these individual trials, with the majority of studies conducted within two labs, utilizing standardised extracts at the same or similar doses.

## Novel Caffeine/Phytochemical Combinations

It is quite possible that co-administration of caffeine alongside other psychoactive phytochemicals, including those noted above, will result in additive or interactive effects with regard to the nervous system. This may be either as a consequence of bi-directional modulation of any neurotransmission effects or due to caffeine’s multifarious effects on the absorption, distribution, metabolism and excretion of other compounds [34]. In this regard it is notable that, for instance, monoterpenes [232, 233] and triterpenes, including ginsenosides [234, 235], alongside many other phytochemicals, are substrates for the same CYP450 enzymes as caffeine (e.g., CYP1A2). This mechanism alone may modulate either of the components’ bioavailability and predispose their combination to have different, potentially greater effects than either component alone. As an example of this, a study in rats found that ginsenosides and caffeine had a synergistic effect with regard to antidepressant effects [236]. However, to date, the question of caffeine’s interactive properties with the phytochemicals that are not found in caffeine synthesising plants is largely unexplored.

## Conclusions and Future Directions

Globally, caffeine is the most widely consumed psychoactive compound and ergogenic aid. When taken in a purified form in a research context caffeine has reasonably well delineated ergogenic and psychological effects. However, in mental performance terms, these effects do not consistently extend beyond improving attention, including psychomotor function and vigilance. Caffeine alone has little impact on the other cognitive domains intrinsic to aspects of sporting performance. Additionally, research almost exclusively investigates the effects of single, acute doses of caffeine, and so the extent to which these findings can be applied to real-world scenarios of repeated daily consumption is arguably limited. In the real world, athletes and participants in sport typically consume caffeine alongside a complex mixture of other potentially bioactive compounds, either in the form of products derived from caffeine-synthesising plants or as an additive to multi-ingredient products. Given caffeine’s many potentially interactive mechanisms of action, it is unsurprising that the psychological effects of these complex combinations of potentially bioactive compounds often exceed those that would be expected from caffeine alone.

As an aside, this does raise important questions of safety and, whilst the trials included within this narrative review report no serious adverse events, it would be remiss of this review not to highlight the continued need to question the potential unanticipated effects of combining different compounds within one product and the effects of cumulative doses of the ingredients. The individual doses of each compound alone may not result in an adverse event, but there is scope for unforeseen negative effects following their combination. This is especially concerning when one considers that caffeine may enhance the absorption and distribution of other co-consumed ingredients (and vice versa), and so, whilst doses might seem safe in isolation, their enhanced pharmacokinetics may evince unsafe psychophysiological effects. As an example, this has been reported to a small extent with over-consumption of energy drinks in adolescent groups [237].

Controlled trial evidence in humans has directly confirmed functional interactions between caffeine and polyphenols, l-theanine and taurine. Additionally, high polyphenol extracts from several caffeine-synthesising plants with low levels of caffeine engender broader benefits to mental performance than expected from caffeine, even at much higher doses. This is certainly the case for high-flavanol cocoa and guaraná extracts, and high-CGA coffee berry. In the case of high-flavanol cocoa extracts the benefits to psychological functioning are evident when directly compared to caffeine-matched control treatments. This gives a clear indication of the added value of cocoa-flavanols, but does not disentangle any interactions in the combination.

Herein lies the problem for this research area. Very few of the many controlled trials assessing the psychological or ergogenic effects of caffeinated products have been designed with the requisite comparator arms to disentangle the interactive effects of caffeine. Future trials should focus both on combination products and their caffeine content alone and/or the combination minus caffeine. Ideally, studies could also instigate a full fractionation of all possible permutations of combination products. Further, trials rarely investigate both the acute and chronic effects of consuming caffeine alongside these additional ingredients. It may therefore be the case that where unconvincing acute effects of these combinations do exist, that longer term administration may result in more pronounced, or at least different, effects. Given the often sport/activity-specific effects of the compounds covered in this narrative review, it may also be the case that research has yet to home in on the most effective matching between physical performance requirements, caffeine and its co-consumed compounds. As such, where these unconvincing effects exist, it is probably too premature to discount them entirely. As an additional caveat, future research should undoubtedly be more representative of non-male participants.

It seems likely that consuming pure anhydrous caffeine is the most impoverished method of delivering caffeine for the enhancement of either physical or psychological functioning. However, more research is needed on a number of fronts. First, to disentangle the contributions of caffeine and the non-caffeine bioactive compounds in caffeinated products. Second, to establish the optimal level of caffeine in caffeinated products, including the potential for additional caffeine to further enhance the functional benefits of low caffeine extracts. Finally, to explore the potential for caffeine to potentiate the benefits seen following multifarious other psychoactive phytochemicals that have not been meaningfully combined with caffeine to date.
